# Parametric Excitation of Optomechanical Resonators by Periodical Modulation

**DOI:** 10.3390/mi9040193

**Published:** 2018-04-18

**Authors:** Jianguo Huang, Muhammad Faeyz Karim, Jiuhui Wu, Tianning Chen, Aiqun Liu

**Affiliations:** 1 School of Mechanical Engineering, Xi’an Jiaotong University, Xi’an 710049, China; jghuang@ntu.edu.sg (J.H.); ejhwu@mail.xjtu.edu.cn (J.W.); tnchen@mail.xjtu.edu.cn (T.C.); 2 School of Electrical & Electronic Engineering, Nanyang Technological University, Singapore 639798, Singapore; faeyz@ntu.edu.sg

**Keywords:** optomechanics, extended working range, periodical modulation

## Abstract

Optical excitation of mechanical resonators has long been a research interest, since it has great applications in the physical and engineering field. Previous optomechanical methods rely on the wavelength-dependent, optical anti-damping effects, with the working range limited to the blue-detuning range. In this study, we experimentally demonstrated the excitation of optomechanical resonators by periodical modulation. The wavelength working range was extended from the blue-detuning to red-detuning range. This demonstration will provide a new way to excite mechanical resonators and benefit practical applications, such as optical mass sensors and gyroscopes with an extended working range.

## 1. Introduction

The ability to optically control mechanical resonators is the key requirement of many technological and fundamental advances in physical and engineering field [1,2,3,4]. The recent development of optomechanics—which utilizes the photon-phonon coupling in nanoscale photonic structures—has provided a new means to manipulate and measure mechanical resonators [5,6,7,8]. The retarded optical force within an optical cavity can be used to cool the mechanical resonators into quantum ground state or to amplify the mechanical resonators into self-oscillation. Recently, several groups have cooled the mechanical motion close to the quantum ground state both in the microwave domain [9] and in the optical domain [10]. For practical applications, optomechanical excitation is attracting more and more attention due to its great technological value. Nowadays, plenty of promising applications have been demonstrated, such as radiofrequency optomechanical resonators [11], nonvolatile optical memory [12], wavelength routing [13], optomechanical sensing [14], and so on.

**Figure 1:**
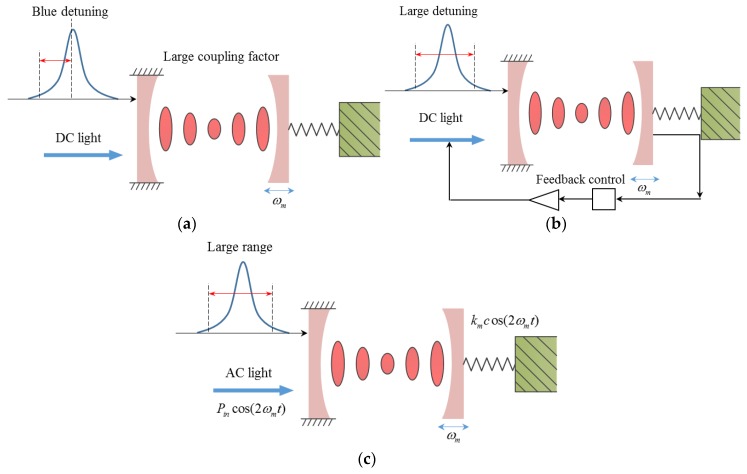
Schematic of different methods for excitation of optomechanical resonators. (**a**) Passive backaction between cavity and mechanical cantilever by direct current (DC) light using optical anti-damping effect; (**b**) active feedback control by DC light using optical anti-damping effect; (**c**) modulated light at twice the mechanical frequency using optical spring effect.

Conventional excitation of optomechanical resonators relies on passive backaction between mechanical resonators and optical cavities [15] or active feedback control [16], which are shown in Figure 1a,b. The optical anti-damping effect is used in the passive backaction method, in which the optical anti-damping is large enough to compensate the intrinsic mechanical damping to excite mechanical resonators [14]. Due to optical anti-damping depending on input optical wavelength, the working range is limited in the blue-detuning range, in which the input wavelength is smaller than the cavity resonance wavelength. Complex off-chip control systems are needed in the active feedback control method, although it can work in the red-detuning range [17,18,19]. From the aspects of device applications, it is desirable to extend the working range in a single chip without complex control systems. Even though manipulating the optomechanical system by an amplitude-modulated light has been proposed both in theory and experiments [20,21,22,23], the parametric excitation has not been demonstrated.

In this study, excitation of optomechanical resonators by periodically modulating optical spring at twice the natural frequency was experimentally demonstrated, which is shown in Figure 1c. This method is achieved by utilizing optical spring rather than optical anti-damping effects. It can break the wavelength limit by extending the working wavelength from the blue-detuning to red-detuning range. This demonstration will provide a new way to excite mechanical resonators and benefit practical applications, such as optical mass sensors and gyroscopes with an extended working range [24,25,26,27].

**Figure 2:**
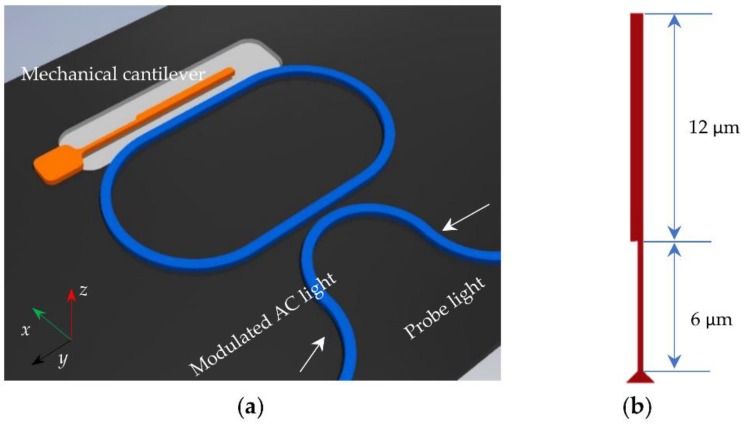
(**a**) The schematic illustration of modulated method by alternating current (AC) light using optical spring effect for mechanical excitation; (**b**) the size of mechanical cantilever.

The proposed excitation of optomechanical resonator by periodical modulation consists of a mechanical cantilever, a racetrack optical cavity, and a curved bus waveguide as shown in Figure 2. Mechanical cantilever is located at the side of the racetrack optical cavity with a gap of 200 nm to build optical coupling between cantilever beam and racetrack cavity. The mechanical cantilever is released from the substrate with a gap of 500 nm and can vibrate in the *x*-direction and *z*-direction, corresponding to fundamental and second mechanical modes. The mechanical cantilever consists of two parts: one part has a cross-section of 200 nm × 340 nm with a length of 6 µm and the other part has a cross-section of 450 nm × 340 nm with a length of 12 µm. The curved bus waveguide is coupled to the racetrack optical cavity with a gap of 200 nm. 

When the pump light is coupled into the racetrack optical cavity through the bus waveguide, optical force due to the evanescent wave overlapping is generated between the mechanical cantilever and racetrack optical cavity. When the pump light is amplitude modulated at twice the mechanical frequency, the alternating optical force will drive the mechanical cantilever and amplify the mechanical vibrations, which are known as parametric excitations.

The pump light was amplitude modulated in the experiment, resulting in the periodical modulation of optical light in the cavity. The intracavity mode amplitude *a* and the coupled mechanical resonator are described as

(1) $$\dot{a}(t)=\left(i(\Delta -g_{om}x)-\kappa /2\right)a(t)+\kappa \sqrt{n}/2$$

(2) $$m_{eff}\ddot{x}+m_{eff}\gamma_{m}\dot{x}+m_{eff}\omega_{m0}{}^{2}x=\hbar g_{om}a^{*}a$$

where Δ = *ω*_0_ − *ω* is the frequency detuning, *ω* is the driving laser frequency, *ω*_0_ is the optical cavity resonance frequency, *g*_om_ is the optomechanical coupling, *κ* is the cavity linewidth, *n* is the cavity photon number when the detuning is zero $n=4P_{in}\kappa_{e}/\kappa^{2}\hbar \omega$, *κ_e_* is the external cavity linewidth and $P_{in}=P_{0}+P_{ac}\cos(2\omega_{m0}t)$ is the optical input with static power $P_{0}$ and alternating power $P_{ac}$, *ω*_*m*0_ is the mechanical frequency, *m*_eff_ is the effective mechanical mass, $\gamma_{m}$ is the mechanical damping factor, *x* is the displacement of mechanical resonator.

To simulate the response of the coupled Equations (1) and (2), the equations can be generalized and reduced to

(3) $$\dot{\tilde{a}}(\tilde{t})=\left(i(\tilde{\Delta }-{\tilde{g}}_{om}\tilde{x})-\tilde{\kappa }/2\right)\tilde{a}(\tilde{t})+\tilde{\kappa }\sqrt{1+\varepsilon \cos(2\tilde{t})}/2$$

(4) $$\ddot{\tilde{x}}+{\tilde{\gamma }}_{m}\dot{\tilde{x}}+\tilde{x}=J\tilde{a}{\tilde{a}}^{*}$$

where the frequencies are normalized by $\omega_{m0}$, $\tilde{t}=\omega_{m0}t$, $\tilde{x}=g_{om}x/\omega_{m0}$, $\tilde{a}=a/\sqrt{n_{0}}$, $n_{0}=4P_{0}\kappa_{e}/\kappa^{2}\hbar \omega$, $\tilde{\Delta }=\Delta /\omega_{m}$, ${\tilde{g}}_{om}=1$, $\tilde{\kappa }=\kappa /\omega_{m0}$, ${\tilde{\gamma }}_{m}=\gamma_{m}/\omega_{m0}$, $\varepsilon =P_{ac}/P_{0}$ is the modulation strength, and $J=4\hbar n_{0}g_{om}{}^{2}/m_{eff}\omega_{m0}{}^{3}$ is the normalized optomechanical coupling strength.

**Figure 3:**
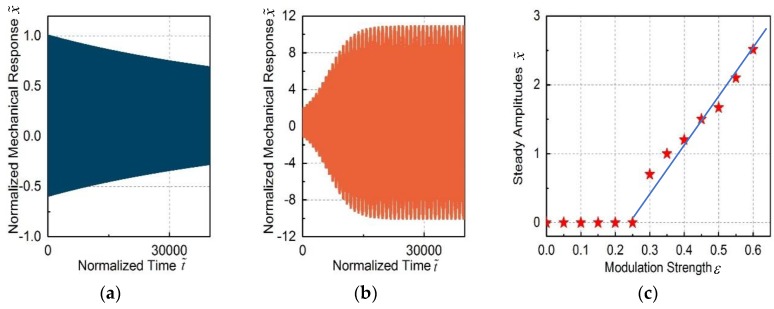
The theoretical simulation of the normalized mechanical response under the periodical modulation. (**a**) The mechanical oscillator is not excited at *ε* = 0; (**b**) the mechanical oscillator is excited at *ε* = 1; (**c**) the steady amplitudes as a function of the modulation strength.

To get the physical insight of this model and simplify the simulation complexity, the parameters are set as $\tilde{\Delta }=-3$, $\tilde{\kappa }=25$, ${\tilde{\gamma }}_{m}=1.2\times 10^{-3}$, J=0.22, ${\tilde{g}}_{om}=1$. By setting ε=0, Equations (3) and (4) are reduced to the conventional optomechanical coupled oscillators. The simulated response of the mechanical oscillators is shown in Figure 3a, in which the mechanical oscillator is not excited. However, the mechanical oscillator is excited into self-oscillation by setting ε=1 with other parameters the same, which is shown in Figure 3b. It can be seen that the modulation can effectively excite the mechanical resonators into self-oscillation even though the passive feedback is too small.

The steady response of the mechanical amplitudes as a function of modulation strength is shown in Figure 3c. When the modulation strength is smaller than ε=0.25, the mechanical oscillators are not excited. Above a certain threshold power ε>0.25, the mechanical oscillators are excited into self-oscillation and the amplitudes increase approximately linearly with the modulation strength. This can be explained as follows: as the mechanical oscillation are excited into self-oscillation, the amplitudes are saturated and increase linearly with the power. 

## 2. Fabrication and Experimental Setup

The fabrication process of the optomechanical resonators is described in this section, which is based on the silicon nanophotonic processes [28,29]. The racetrack optical cavity had a footprint of 25 µm × 20 µm and was fabricated in the silicon-on-Insulator (SOI) wafer, which had a 2 µm box layer between the 340 nm silicon structure layer and substrate. The optomechanical resonator was patterned by deep-ultraviolet lithography and followed by plasma dry etching. Note that a hard mask process was adopted to ensure accurate etching because the photoresist is too thick to withstand reactive ion etching [30]. When the etching depth is above 100 nm, the hard mask can help to protect the etching profiles in order to ensure the linewidth and sidewall surface roughness.

**Figure 4:**
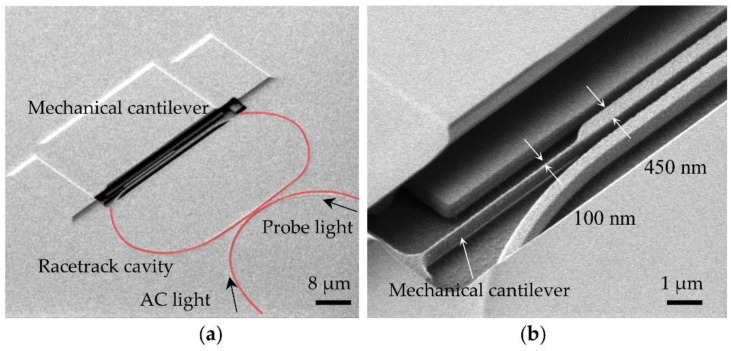
(**a**) scanning electron microscope (SEM) image of the fabricated device; (**b**) zoom-in view with the width labeled.

A hard mask made from a 70 nm thin layer of SiO_2_ was deposited on the surface of wafer by low-temperature plasma-enhanced chemical vapor deposition (PECVD). Subsequently, a 90 nm bottom anti-reflective coating (BARC) and 700 nm photoresist (PR) was dispensed on the surface of the hard mask. The bottom anti-reflective coating is also a must to reduce the standing wave effect from the lithography to ensure the linewidth. Another important issue is the hydrogen fluorider release process, which is used to make the silicon structures movable in the desired directions. The hydrogen fluorider vapor is good choice to release the structures because it only reacts with SiO_2_ but not with silicon waveguide. These unique characters can leave the etched waveguide with a good profile and a smooth surface without causing the stiction problems. More importantly, the gap between the silicon and substrate can be controlled by choosing different etching times. The etching rate is estimated as 30 nm/min in our structures. A scanning electron microscope (SEM) image of the fabricated device is shown in Figure 4a,b. It should be noted that the estimated width of the fabricated mechanical cantilever is 100 nm, which is smaller than the original design of 200 nm. This is due to imperfect fabrication, such as lithography or etching, in which an unwanted deviation can occur. 

**Figure 5:**
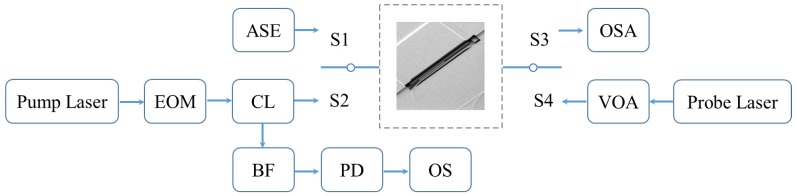
The schematic of the experimental setup. Abbreviations: EOM, electro-optical modulator; CL, optical circulator; VOA, variable optical attenuator; BF, bandpass filter; PD, photon detector; OS, oscilloscope; ASE, amplified spontaneous emission light; OSA, optical spectrum analyzer; S1–S4, optical switch.

The experimental setup is shown in Figure 5. In the experiment, the probe light was used to measure the mechanical cantilever in the racetrack optical cavity and pumped in a different direction from the pump light to avoid influencing the pump light. The transmission spectrum of the optical cavity was firstly measured by a broadband light from a 12 dBm ASE light source (Amonics ALS-CL-13, Kowloon, Hong Kong, China) and then detected by an optical spectrum analyzer (OSA) (AQ6370D, Yokogawa Test & Measurement Corporation, Tokyo, Japan) by switching S1 and S3. The resolution of the OSA was 0.02 nm. By switching S2 and S4, the mechanical response was measured. The pump light from a tunable laser (Santec TSL 510, Hackensack, NJ, USA) was modulated by an electrical-optical modulator and the frequency was controlled by a programmable function generator (PM 5193, Philips, Amsterdam, The Netherlands). The power of the probe light from a tunable laser (Santec TSL 510, Hackensack, NJ, USA) was controlled by the variable optical attenuator. The probe light was separated from the pump light by the bandpass filter and was received by the photodetector (FPD 510, Menlo Systems GmbH, Planegg, Germany), in which the power spectral density of the mechanical oscillation in the frequency domain and time domain was shown in the oscilloscope (MDO4104B-3, Tektronix, Inc., Beaverton, OR, USA). The device was measured under a high-vacuum chamber (2 × 10^−6^ mBar).

## 3. Experimental Results and Discussions

### 3.1. Optical Characterization of the Racetrack Optical Cavity

**Figure 6:**
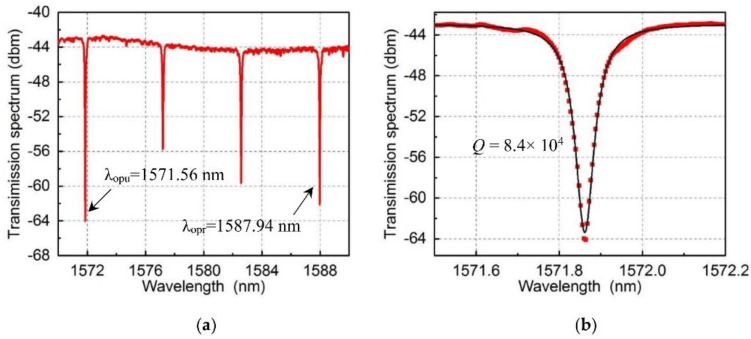
(**a**) Transmission spectrum of the optical racetrack cavity showing resonance at different optical wavelengths. Resonance wavelength at 1571.86 nm was used as the pump light channel and resonance wavelength at 1587.94 nm was used as the probe light channel. (**b**) The zoom-in transmission spectrum at 1571.86 nm with a Lorenz fitting, which shows the optical quality factor, is *Q* = 8.4 × 10^4^.

In the experiment, the transmission spectrum of the racetrack resonator was firstly characterized by a low-power broadband light. Figure 6a shows the output light from the grating coupler.

The optical resonance wavelength was separated by the free spectral range (FSR) with a range of 5.34 nm. In order to separate the pump and probe light, resonance wavelength at 1571.86 nm was used as the pump light channel and resonance wavelength at 1587.94 nm was used as the probe light channel. The transmission spectrum at 1571.86 nm with a Lorenz fitting is shown in Figure 6b. The optical quality factor is 8.4 × 10^4^ with a dip of 20 dB.

### 3.2. Optical Characterization of the Mechanical Cantilever

**Figure 7:**
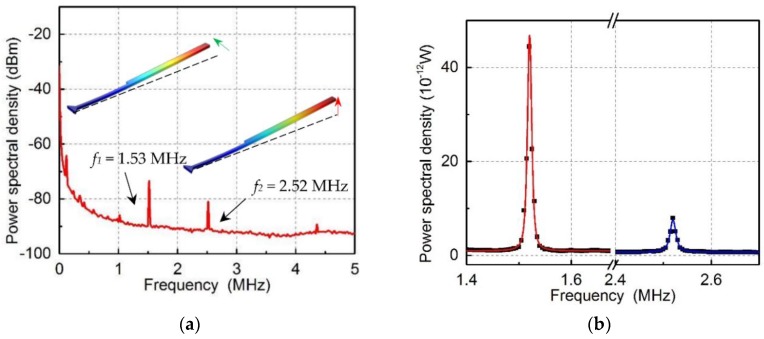
(**a**) The power spectral density of the measured signals, showing the mechanical vibration motions. The inset is the finite element simulation of the two mechanical vibration modes, which correspond to *x* and *z* direction. (**b**) The zoom-in power spectrum density of two mechanical oscillators.

After the characterization of the optical racetrack cavity, another important issue is to characterize the vibration mode of the mechanical cantilever. In the experiment, a probe light with a power of 20 µW at wavelength *λ_pr_* = 1587.90 nm was coupled to the cavity and the output light was analyzed by the oscilloscope. The power spectral density of the mechanical motion is shown in Figure 7. Since the mechanical cantilever can vibrate in the *x* and *z* direction, two mechanical modes are 1.53 MHz with mechanical linewidth of 1 kHz, and 2.52 MHz with mechanical linewidth of 1.5 kHz, respectively. The finite element simulation of the mechanical modes is shown in the inset figures. It should be noted that the residual stress due to the fabrication will result in a shift of the practical mechanical resonance frequency.

### 3.3. Excitation of the Mechanical Cantilever

**Figure 8:**
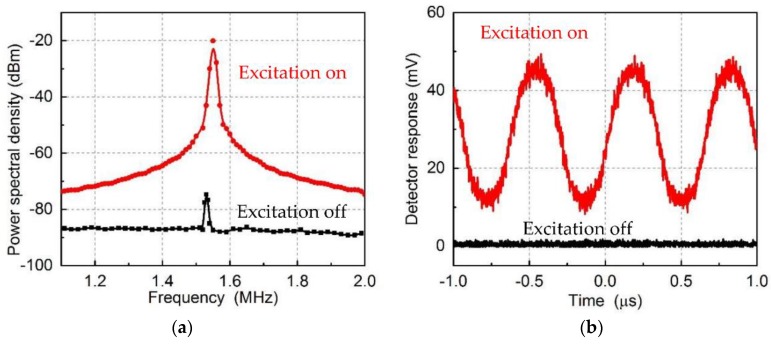
(**a**) The power spectral density of the measured signals, showing the thermal mechanical noise (black curve) and amplified mechanical motions (red curve) when the pump light is in the state of off and on separately; (**b**) the time domain traces showing the original mechanical vibrations and amplified vibrations separately.

In order to demonstrate the parametric excitation of mechanical cantilever, a modulated pump light with a power of 200 µW at wavelength *λ_pu_* = 1571.50 nm was coupled to the racetrack optical cavity to excite the mechanical cantilever. When the modulated frequency is twice the mechanical frequency *ω_p_*/2*π* = 3.06 MHz, the mechanical cantilever is excited, which is shown in Figure 8a. The black and red curve shows the original thermal mechanical vibrations and amplified vibrations separately. It can be seen that the power spectral density is greatly excited from −79 dBm to −20 dBm. This excitation can also be demonstrated in the time domain, which is shown in Figure 8b, in which the mechanical cantilever is excited by the pump light. The peak in Figure 8a is −25 dBm, which corresponds to 12.5 mV rms (35 mV pp) in Figure 8b. The peak in black curves is thermal motion of the cantilever.

**Figure 9:**
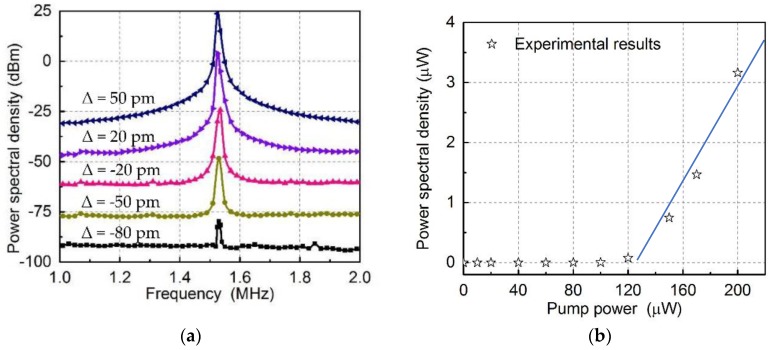
(**a**) The power spectral density of the measured signals when the wavelength detuning is from −80 pm to 50 pm (curves are vertically offset for clarification); (**b**) the peak value of power spectral density at the mechanical resonance frequency versus the pump power.

Then, the wavelength of the pump light was changed to demonstrate the extended working range. The detuning of the pump light Δ = *λ_pu_* − *λ_opu_* was swept from −80 pm to 50 pm at fixed a power of 200 µW to observe the parametric excitation, which is shown in Figure 9a. It can be seen that the working range is extended from the blue-detuning to red-detuning region with a range of 130 pm. At the same time, increasing the modulation strength can enhance the excitation. In the experiment, the power of the pump light was gradually increased from 0 to 200 µW at fixed −60 pm to observe the effect of the modulation strength on the excitation. The detailed experimental results are shown in Figure 9a. It should be noted that the results shown in Figure 9a,b were obtained in different conditions: one is for fixed optical power, while the other one is for fixed optical wavelength. By increasing the power of pump light, the mechanical motion was gradually amplified. The relationship between the peak value and the pump power is shown in Figure 9b with a fitting curve. Even though amplitudes increase with the optical power, larger optical power will result in the instability of the system due to the thermo-optical effects of the silicon structures. The largest amplitude of the mechanical oscillator is estimated as 5 nm. Since the optomechanical coupling factor is *g_om_*/2*π* = 450 MHz/nm, the cavity frequency shift is 2*π* × 2.25 GHz, which is comparable to the cavity linewidth 2*π* × 2.27 GHz.

It can be seen from Figure 9b that the power spectral densities are not linear with the optical power. Here, we give a rough explanation for this nonlinearity. When the mechanical oscillators are excited, the mechanical nonlinearity starts to play a role. In particular, our theoretical simulation is based on small linear approximation, so when the mechanical oscillation is large, the nonlinearity [31,32] should be taken into account.

## 4. Conclusions

In this study, the excitation of optomechanical resonators by periodical modulation was experimentally demonstrated. A mechanical cantilever is excited by the modulated optical field in an optical resonator with an optical quality factor of 8.43 × 10^4^. The working wavelength of the pump light is extended from blue-detuning to red-detuning region with a range of 130 pm. The excitation is enhanced by increasing the optical power. Compared with conventional mechanical excitation induced by the passive back action or active feedback control, this method provides a simple and effective approach [33,34,35]. This demonstration will benefit the optical mass sensor based on the parametric excitation and be useful for improving performance in practical applications, such as gyroscopes with an extended working range.
